# Recent Advances in Allergic Rhinitis: A Narrative Review

**DOI:** 10.7759/cureus.68607

**Published:** 2024-09-04

**Authors:** Megha Tidke, Pramod T Borghare, Piyush Pardhekar, Yugandhara Nasre, Kavita Gomase, Minakshi Chaudhary

**Affiliations:** 1 Otolaryngology, Mahatama Gandhi Ayurveda College, Hospital and Research Centre, Wardha, IND; 2 Otolaryngology, Bhausaheb Mulak Ayurved Mahavidyalaya &amp; Medical Science &amp; Research, Nagpur, IND; 3 Obstetrics and Gynaecology, Smt. Radhikabai Meghe Memorial College of Nursing, Datta Meghe Institute of Higher Education and Research, Wardha, IND; 4 Nursing, Shalinitai Meghe College of Nursing, Datta Meghe Institute of Higher Education and Resesrach, Wardha, IND

**Keywords:** precision medicine, nasal polyps, biologics, immunotherapy, allergic rhinitis

## Abstract

Allergic rhinitis (AR) is a prevalent chronic respiratory condition characterized by nasal inflammation, sneezing, congestion, and itching, significantly impacting quality of life. Over recent years, considerable advancements have been made in understanding the pathophysiology, diagnosis, and management of AR. This narrative review aims to synthesize these recent developments, providing a comprehensive overview of key areas. Emerging insights into AR pathophysiology have elucidated the complex interplay between genetic predisposition, environmental factors, and immune system dysregulation. Notably, the role of the epithelial barrier and the microbiome in AR pathogenesis has garnered increasing attention, offering potential targets for novel therapies. Advances in diagnostic technologies, such as component-resolved diagnostics and molecular allergology, have enhanced the precision of allergy identification, enabling more personalized treatment approaches. In terms of management, significant progress has been made in pharmacological and non-pharmacological treatments. Novel biologics targeting specific pathways involved in AR, including monoclonal antibodies against immunoglobulin (Ig)E and interleukin (IL)-4/13, have shown promise in reducing symptoms in refractory cases. Additionally, there has been a resurgence in interest in non-pharmacological strategies, including allergen avoidance, immunotherapy, and complementary therapies, which offer holistic options for patient care. The integration of digital health tools and mobile applications in AR management has further empowered patients, allowing for real-time symptom tracking and personalized treatment adjustments. Recent guidelines emphasize a multidisciplinary approach to AR management, promoting integrated care models that involve collaboration between allergists, primary care providers, and other specialists. These guidelines also highlight the importance of patient-centered care, advocating for shared decision-making and tailored treatment plans based on individual patient profiles. In conclusion, the landscape of allergic rhinitis management is rapidly evolving, with ongoing research and innovation paving the way for improved outcomes. This review underscores the importance of staying abreast of these advances to optimize the care and quality of life for individuals affected by allergic rhinitis.

## Introduction and background

Allergic rhinitis (AR) is a widespread immunologic condition marked by inflammation of the nasal mucosa, which occurs due to an immunoglobulin (Ig)E-mediated hypersensitivity reaction to environmental allergens. It impacts a large segment of the global population, with prevalence rates ranging from 10% to 30% in different areas, underscoring its significant public health implications [1]. The condition is divided into two primary types: seasonal allergic rhinitis (SAR) and perennial allergic rhinitis (PAR), depending on when and how long the symptoms occur [2]. Recent advancements in our understanding of the pathophysiology of AR have greatly enhanced diagnostic and therapeutic strategies. Innovations in allergen immunotherapy, such as sublingual and subcutaneous treatments, have shown promising results in reducing symptom severity and minimizing the need for medication.

Furthermore, new biological agents that target specific cytokines involved in the allergic inflammation process, such as anti-IgE and anti-interleukin (IL)-4/IL-13 therapies, have become effective treatment alternatives for patients suffering from moderate to severe AR [3]. Recent technological advancements, such as high-throughput sequencing and bioinformatics tools, have made it easier to identify new sources of allergens and biomarkers for personalized medicine approaches [4]. Additionally, recent studies have emphasized the influence of environmental and genetic factors on the development and worsening of AR, offering fresh perspectives on prevention and management strategies [5].

This narrative review aims to synthesize recent advancements in the understanding and management of allergic rhinitis, focusing on new therapeutic modalities, diagnostic innovations, and the role of environmental and genetic factors. By providing a comprehensive overview of current research and emerging trends, this review seeks to inform clinicians and researchers about the latest developments in allergic rhinitis.

## Review

Search methodology

The methodology for the narrative review on "Recent Advances in Allergic Rhinitis" included a thorough literature search across several electronic databases, such as PubMed, Scopus, and Google Scholar, covering the timeframe from January 2018 to August 2024. We utilized keywords like "allergic rhinitis," "recent advances," "novel therapies," "pathophysiology," "diagnostic technologies," "management guidelines," and "non-pharmacological treatments" in various combinations to find relevant studies. The search was limited to peer-reviewed articles, clinical trials, review papers, and guidelines published in English. We also identified additional references by manually searching the reference lists of critical articles. Studies were chosen based on their relevance to recent developments in the field, mainly focusing on innovations in diagnosis, treatment, and management strategies for allergic rhinitis.

Pathophysiology of allergic rhinitis

Immune Response in AR

Role of immunoglobulin E (IgE) and allergen sensitization: AR is an IgE-induced inflammation of the nasal mucosa that presents with sneezing and/or a runny or blocked nose. AR involves the following immune response: Understanding of AR commences with sensation to allergens such as pollen, dust mites, or pet dander. In the first sensitization, dendritic cells or any other antigen-presenting cells (APC) in the body become activated, internalize the allergens, and then present them to the T helper 2 (Th2) cell. This interaction stimulates Th2 cells to secrete cytokines such as the interleukins, including IL-4, IL-5, and IL-13. These cytokines are essential to call for B cell subclass switch and secretion of allergen-specific IgE [6]. Once produced, they bind the high-affinity functional high-affinity immunoglobulin E receptors (FcεRI) located on mast cell and basophil surfaces, called sensitization. For the second time and in subsequent exposures to the same allergen, it binds to the IgE on these sensitized cells, resulting in degranulation of these cells and release of both preformed and newly formed mediators of inflammation, inclusive of histamine, leukotrienes, and prostaglandins. These mediators are to blame for the typical manifestations of AR, such as sneezing, itching, nasal congestion, and a runny nose [7].

Inflammatory mediators and cytokines involved: The inflammatory response in allergic rhinitis (AR) involves a complex interplay of various cells and cytokines. Besides IgE, other immune cells such as eosinophils, T cells, and epithelial cells play significant roles in this inflammatory process. When allergens are encountered, epithelial cells release thymic stromal lymphopoietin (TSLP), IL-33, and IL-25, which help to boost the Th2 response by activating innate lymphoid cells (ILC2s) and increasing the production of Th2 cytokines [8]. Histamine, released from mast cells, binds to H1 receptors on various cells, causing vasodilation, increased vascular permeability, and mucus production. Leukotrienes (C4, D4, and E4) contribute to bronchoconstriction, mucus secretion, and increased vascular permeability, exacerbating nasal congestion and other AR symptoms. Prostaglandin D2 (PGD2), also released from mast cells, recruits Th2 cells, eosinophils, and basophils to the site of inflammation, perpetuating the allergic response [9]. Cytokines like IL-4 and IL-13 are vital in sustaining the Th2 response and encouraging IgE production. IL-5 is especially significant for the activation and survival of eosinophils, which results in their accumulation in the nasal mucosa, a key feature of chronic AR. Additionally, pro-inflammatory cytokines such as tumour necrosis factor-alpha (TNF-α) and IL-1β help recruit inflammatory cells and enhance the inflammatory response [10].

Genetic and Environmental Factors

Genetic predisposition: AR is a multifactorial disorder with a vital genetic component. Numerous studies have demonstrated that individuals with a family history of atopy are more likely to develop allergic rhinitis. Twin studies have shown a higher concordance rate for allergic diseases in monozygotic twins than in dizygotic twins, indicating a significant genetic influence [11]. Several candidate genes have been implicated in the pathogenesis of AR, including those involved in the immune response, such as the genes encoding for the high-affinity IgE receptor (FCER1A) and the IL-4 receptor (IL4R). Genome-wide association studies (GWAS) have also identified loci associated with AR, highlighting the role of specific genetic variations in the susceptibility to this condition [12].

Influence of environmental factors: Environmental factors play a crucial role in developing and exacerbating allergic rhinitis. Exposure to allergens, such as pollen, dust mites, mould, and pet dander, is a well-known trigger for AR symptoms. Urbanization and the increased prevalence of pollutants, including particulate matter, ozone, and tobacco smoke, have been linked to a higher incidence of allergic diseases [13]. Additionally, climate change has led to longer pollen seasons and higher pollen concentrations, further contributing to the rise in AR cases. The "hygiene hypothesis" suggests that reduced exposure to microbial agents in early childhood, due to improved hygiene practices, may predispose individuals to allergic diseases by altering the immune system's development. This hypothesis is supported by epidemiological studies showing lower rates of AR in individuals from rural areas or those with a history of infections during childhood [14].

Recent Discoveries in AR Pathophysiology

Novel biomarkers: Recent research has identified several novel biomarkers that provide deeper insights into the pathophysiology of AR. Among these, periostin, a matricellular protein, has gained attention due to its role in chronic allergic inflammation. Periostin is upregulated in response to IL-13 and IL-4, key cytokines in Th2-mediated allergic reactions, making it a potential biomarker for chronic AR and a predictor of response to treatment with biologics targeting these pathways [15]. Another promising biomarker is nasal nitric oxide (nNO), which has been explored as a non-invasive marker of nasal inflammation. Elevated levels of nNO have been associated with eosinophilic inflammation in AR patients, reflecting ongoing allergic responses and the potential severity of the condition [16]. Moreover, omics technologies, such as proteomics and transcriptomics, have revealed unique protein and gene expression profiles in AR patients, offering potential biomarkers for disease severity and treatment response [17].

Advances in understanding the cellular and molecular mechanisms: The understanding of cellular and molecular mechanisms underlying AR has significantly advanced in recent years. One major discovery involves the role of epithelial cells in initiating and perpetuating allergic inflammation. Epithelial cells in the nasal mucosa, upon exposure to allergens, release cytokines such as thymic stromal lymphopoietin (TSLP), IL-25, and IL-33, which activate dendritic cells and type 2 innate lymphoid cells (ILC2s). These ILC2s, in turn, produce large amounts of IL-5 and IL-13, driving the differentiation and recruitment of eosinophils and Th2 cells, respectively [18]. Furthermore, the discovery of the role of the epithelial barrier in AR pathophysiology has shed light on how environmental factors, such as pollutants and microbes, may exacerbate allergic responses. Disruption of the epithelial barrier function can lead to increased allergen penetration and heightened immune responses, suggesting new therapeutic targets to restore or enhance barrier integrity [19]. The role of microRNAs (miRNAs) in AR has also emerged as a critical area of research. Specific miRNAs have been found to regulate the expression of genes involved in Th2 differentiation and cytokine production. For example, miR-21 has enhanced Th2 responses by targeting and downregulating IL-12, a cytokine that typically promotes Th1 differentiation, thereby skewing the immune response towards a Th2-dominant profile [20].

Advances in diagnostic techniques

Traditional Diagnostic Methods

Skin prick testing (SPT): One of the widely used, well-established methods for diagnosing specific allergens responsible for AR is the SPT. In the process, small amounts of allergen extract are introduced into the skin with a lancet; generally, the area used is the forearm or the back. After the prick, the test area is then noted for the formation of a wheal and flare reaction, indicating specific IgE antibodies against the tested allergens. Direct measurement of the wheel size will allow for determining the degree of allergic response. SPT is highly sensitive, inexpensive, and gives quick results; therefore, it remains one of the preferred methods for diagnosing AR in clinical practice [21].

Serum-specific IgE testing: In vitro allergy tests determine the presence of allergen-specific IgE antibodies in a patient's blood sample. The test is functional when SPT cannot be performed or is unreliable, for instance, if there is a generalized skin disease, dermatographism, or it is not possible to stop antihistamine therapy. Serum-specific IgE is handy for confirming the results of SPT. The enzyme-linked immunosorbent assay (ELISA), which gives quantitative results and allows allergen sensitivity to be assessed, is the most commonly used method for this. The technique offers the advantages of being safe and allowing for the simultaneous testing of multiple allergens. However, it could be more costly and possibly less sensitive than SPT [22].

Emerging Diagnostic Technologies

Component-resolved diagnostics (CRD): Component-resolved diagnostics are a huge step ahead in diagnosing allergic rhinitis. Component Resolved Diagnosis, or CRD, is much more specific than extract-based tests because it detects specific IgE antibodies of an individual using purified or recombinant allergen components. This helps determine individual proteins in a source of allergens, information on cross-reactive allergens, and an exact diagnosis with subsequent treatment options. For example, CRD has been very useful in pointing out the primary sensitization to actual allergens and cross-sensitization due to homologous proteins in other allergenic sources [22].

Molecular allergology: Molecular allergology is defined as the branch very close to CRD, and diagnostic methods are based on molecular biology principles that evaluate the allergic mechanisms involved in allergic diseases such as AR. This approach uses molecular methods like microarrays and mass spectrometry to detect and quantify certain allergenic proteins. Molecular allergology allows the determination of the specific molecules the patient reacts to, making it easier to tailor the management. It also helps identify the likely severity of the reactions, consequently helping better manage the patients [23].

Imaging techniques: Other recommendations that have also improved allergic rhinitis diagnosis and management include recent inventions in imaging systems. Imaging of the nasal mucosa and surrounding structures through resolutions like MRI (magnetic resonance imaging), CT (computed tomography), and functional imaging of PET (positron emission tomography) has also been incorporated. These techniques enable the quantification of inflammation, structural changes, and severity of the disease processes, which are of significant importance in diagnosing and classifying allergic rhinitis. Further, imaging has been crucial in the institution of directed therapies and analysis of the outcomes in the sufferers experiencing allergic rhinitis [24].

Advances in pharmacological treatments

Antihistamines and Intranasal Corticosteroids

Current pharmacologic control of AR relies heavily on antihistamines and intranasal corticosteroid agents. Newer second-generation antihistamines, including cetirizine and fexofenadine, are less sedating than older first-generation antihistamines and are more effective, safe, and less toxic. Fluticasone and mometasone are examples of intranasal corticosteroids that are well known for their potent anti-inflammatory activity and are used to relieve nasal congestion, rhinorrhea, and sneezing. More recent developments include new delivery systems and dosage forms; for example, intranasal aerosols with better particle size distribution, leading to better mucosa uptake and patient compliance [25].

Biologic Therapies

Biologic therapies have now shown some potential as methods for middle to serious forms of AR, particularly for patients who failed to respond to common treatments. In this area, one of the major advancements has been the approval of omalizumab, an anti-Ig E monoclonal antibody. It is indicated in allergic diseases by competitively inhibiting allergen-IgE interaction and blocking the high-affinity IgE receptor on mast cells and basophils. Clinical trials conducted recently have shown that omalizumab is useful in controlling AR symptomatology and enhancing the QOL of patients with Perennial and Seasonal AR [26].

Immunotherapy

Specific allergen immunotherapy, or AIT, is the only curative treatment of AR. Some of these are as follows; Sublingual immunotherapy (SLIT) tablets that are safer and better than subcutaneous immunotherapy (SCIT). Most of the patients diagnosed with AR have attempted SLIT, and case reports, controlled trials, and meta-analyses have supported its efficacy in terms of symptom and medication decrease [27].

Advances in Combination Therapies

A new trend in the treatment of AR indicates the use of combination therapy to increase the effectiveness of pharmacological interventions. When two drugs act together, the therapeutic effect of one enhances that of the other - a phenomenon called synergism; two such examples include a combination of antihistamine with intranasal corticosteroids and LTRAs combined with antihistamines are even more effective than when used singly. Subsequent research has also demonstrated that such combinations effectively lower nasal and ocular manifestations, especially in severe diseases [28].

Advances in non-pharmacological treatments

Allergen Avoidance Strategies

Allergen avoidance remains a cornerstone in managing AR, particularly in cases where the allergen is identifiable, and exposure is manageable. Recent advancements have emphasized personalized approaches to allergen avoidance, integrating environmental controls and patient education. Strategies such as using high-efficiency particulate air (HEPA) filters, minimizing indoor humidity, and implementing rigorous cleaning regimens to reduce dust mite exposure have shown promise in reducing symptom severity [29]. Additionally, digital tools and apps that help patients monitor allergen levels in real-time have enhanced the practicality and effectiveness of allergen avoidance strategies [30].

Alternative and Complementary Therapies

Alternative and complementary therapies have gained traction as adjunctive treatments for AR. Among these, acupuncture has been the subject of recent research, with several studies suggesting that it may provide symptomatic relief through modulation of the immune system and reduction of inflammatory markers [31]. Herbal remedies, such as butterbur (Petasites hybridus), have also shown potential in clinical trials, demonstrating efficacy comparable to conventional antihistamines but with fewer side effects [32]. Moreover, probiotics have been explored for their role in modulating the gut microbiota and influencing systemic immune responses, with some studies indicating a reduction in AR symptoms following probiotic supplementation [33].

Immunomodulatory Approaches

Recent advances in immunomodulatory approaches for AR focus on modifying the immune system’s response to allergens. Sublingual immunotherapy (SLIT) continues to evolve, with newer formulations aiming to improve patient adherence and outcomes [34]. Studies have explored biologics, such as monoclonal antibodies targeting specific immune pathways, as potential therapies for severe AR. Omalizumab, an anti-IgE antibody, has shown efficacy in reducing symptoms and the need for pharmacotherapy in patients with difficult-to-treat AR. Additionally, research into peptide immunotherapy, which involves using allergen-derived peptides to induce tolerance, represents a novel frontier in AR treatment [35].

Advances in management and guidelines

Recent Guidelines and Consensus Statements

According to recent guidelines and consensus statements, allergic rhinitis management is based on a multidisciplinary intervention that incorporates pharmacological, nonpharmacological, and lifestyle interventions. The 2022 update of the Allergic Rhinitis and its Impact on Asthma guidelines emphasises individualised treatment plans to consider severity, duration of symptoms, and comorbidities. It should be customized according to patient preference. It is also suggested in the guidelines that the management of the patient should follow a stepwise approach and that treatment begins with avoidance of allergens and progress to INCS and, if necessary, the addition of antihistamines and LTRA for more severe cases in pharmacotherapy. The ARIA guidelines also emphasize monitoring the efficiency of treatment and compliance, which should be done through regular follow-up so that adjustments in the management plan can be made whenever required [36].

Integrated Care Models

The development of different integrated models of care concerning allergy rhinitis management has been emphasised to achieve better patient health outcomes, optimising resource use. They consider joining forces with allergists, primary care physicians, pharmacists, and other professionals with collaborative and continuous care. One example is ARIA-ICP, which creates an entirely digital care pathway by using digital tools such as mobile apps and telemedicine to monitor the symptoms, treatment compliance, and environment in real time. This allows for timely adjustments in the management and thus enhances patient engagement with their care [37].

Patient-Centered Care (PCC)

PCC is emerging as the cornerstone of effective management in allergic rhinitis. PCC entails tailoring the treatment to individual patients regarding their basic needs, preferences, and values, eventually enhancing patients' autonomy and satisfaction. New studies demonstrate that the approaches of PCC mean better adherence to therapy, better control of symptoms, and higher patient satisfaction compared with traditional models of care [38].

Future directions in allergic rhinitis research

Future studies on allergic rhinitis should be oriented to the genetic and epigenetic mechanisms of inter-individual differences in susceptibility and variable treatment responses. Elucidation of the role of the microbiome and searching for new biomarkers for diagnosis at presymptomatic stages, or those that could help predict the response to treatment, may improve patients' outcomes significantly in AR. A more personalized therapy in this regard would be the development of advanced biologics and immunomodulatory strategies and the concurrent development of digital health tools for monitoring and management in AR.

## Conclusions

Improvements in allergic rhinitis have substantially improved the current understanding of its pathophysiology, diagnosis, and management. Such innovative research has been done on the mechanisms that drive AR, helping develop more precise diagnostic tools and personalized treatment approaches. Several new therapies, such as novel pharmacological agents, immunomodulatory strategies, and non-pharmacological interventions, also show their potential to get better patient outcomes. As guidelines evolve to embrace these advancements, integrated care models and patient-centred approaches are increasingly being placed at the heart of AR management, facilitating more effective and tailored care to individuals affected by this ubiquitous condition.
